# The impact of the covid-19 pandemic on the risk of social stigma

**DOI:** 10.1192/j.eurpsy.2022.2253

**Published:** 2022-09-01

**Authors:** M.A. Sacco, C. Scalise, A. Zibetti, V. Aquila, L. Abenavoli, P. Ricci, I. Aquila

**Affiliations:** 1 Institute of Legal Medicine, Medical And Surgical Science, Catanzaro, Italy; 2 University Magna Graecia of Catanzaro, Medical And Surgical Sciences, Catanzaro, Italy

**Keywords:** discrimination, stigma, Covid-19

## Abstract

**Introduction:**

Social stigma indicates a process of negative connotation of a person which results in discrimination. The victim of stigma experiences a condition of social exclusion that negatively affects his relationships. The COVID-19 pandemic has spread the fear of being “contaminated”, which has led to the discrimination of a part of population.

**Objectives:**

The purpose of this work is to analyze which people have suffered from stigma due to the COVID-19 pandemic by examining the negative effects on their health during this period.

**Methods:**

A literature review of peer-reviewed articles was performed on Pubmed NCBI database by inserting the keywords: *stigma and COVID-19* in the period 2020-2021.

**Results:**

The data showed that the categories most at risk were positive patients and their families; healthcare workers in COVID-19 wards; Asian people. Discrimination has included avoidance attitudes, physical or verbal abuse, hypersurveillance in public places. Negative effects on victims included anxiety, depression, feelings of rejection and shame, self-harm and suicide. COVID-19 patients attempted to hide the disease by avoiding access to hospital; health personnel developed risk of burnout; Asian restaurants experienced a drop in reservations, even after quarantine period.

**Conclusions:**

Social stigma is a public health problem and greater efforts are mandatory to reduce it including correct information, with the help of social and mass media; social interventions aimed at generating empathy; avoiding the use of negative language focused on stereotypes that could generate fear or discrimination. Such interventions are crucial to reduce discrimination in such a fragile period as COVID-19 pandemic.

**Disclosure:**

No significant relationships.

